# Measuring interdisciplinarity in clinical practice with IPC59, a modified and improved version of IPC65

**DOI:** 10.1371/journal.pone.0197484

**Published:** 2018-07-06

**Authors:** Thomas G. Poder, Nathalie Carrier, Suzanne K. Bédard

**Affiliations:** 1 UETMIS, CIUSSS de l’Estrie–CHUS, Sherbrooke, QC, Canada; 2 CRCHUS, CIUSSS de l’Estrie–CHUS, Sherbrooke, QC, Canada; University of Antwerp, BELGIUM

## Abstract

**Rationale:**

Interdisciplinarity is considered a key concept in the management of complex cases in healthcare. However, working in interdisciplinary teams requires the integration of many concepts and a large amount of effort. To help healthcare managers and professionals identify the strengths and weaknesses of their interdisciplinary team and to ensure its continuous improvement, we developed a tool called the IPC65.

**Objective:**

The purpose of this study was to test the reliability and validity of the IPC65.

**Methods:**

Based on a comprehensive review of the literature and qualitative and quantitative assessments, the IPC65 was developed. In this study, the analysis was based on 392 healthcare professionals and managers from short-term care settings who provided valid responses throughout the province of Quebec in Canada. Descriptive statistics, Cronbach’s alpha values, and inter-item correlations were measured, and a principal component analysis (PCA) was conducted. Item discrimination was used to provide an improved version of the IPC65.

**Results:**

The IPC65 showed good statistical results. The discriminant procedure provided the basis for shortening and improving the IPC65 to form the IPC59. Cronbach’s alpha values ranged from 0.857 to 0.967 in IPC59, demonstrating very good reliability for all four dimensions. The PCA showed good validity.

**Conclusion:**

The IPC59 can be used to assess the degree of integration of key concepts leading to interdisciplinarity.

## Introduction

For many years, the establishment of interdisciplinary teams has been increasingly encouraged as a goal of patient-centered care [1–4]. The general principle of interdisciplinary healthcare is to have professionals from different disciplines work in synergy to provide efficient and quality care to patients [5–8]. However, its implementation is complex, involves different key concepts and is time-consuming [3,9]. For example, teams must learn to work together, develop a good working climate and develop confidence in one another [10].

To date, many tools and indicators have been developed to measure the degree of integration within the health system and across sectors [11–12], but the specific point on how this integration performs within an interdisciplinary team remains largely unexplored. In this setting, we created an initial questionnaire, the IPC65 (French acronym for Interdisciplinarity in Clinical Practice 65 items), to provide health professionals with a tool that allowed them to measure their degree of integration of the concepts leading to interdisciplinary teams in clinical practice and thus to identify their strengths and weaknesses in order to strengthen their effectiveness in the provision of care and quality treatment [13]. This initial questionnaire was created by considering the integration of 4 dimensions in the care process as described by Contandriopoulos et al. [14]: normative integration, functional integration, clinical integration and care integration.

To start the process of creating the IPC65, an exhaustive literature review was conducted to identify over 150 variables that characterized the functioning and effects of a care team working in interdisciplinarity. After several stages of rigorous validation by experts in interdisciplinarity for the questionnaire’s design and content, including a test-retest analysis and qualitative validation, a first version of the questionnaire with 99 items was created [13]. Then, a quantitative assessment to measure the questionnaire’s internal consistency and construct validity led to a new version with 65 items [13]. This initial assessment was conducted in a cohort of 265 healthcare professionals in Quebec, Canada. The IPC65 was statistically validated with data from this cohort by excluding 34 of the 99 items from the questionnaire. However, some items on the IPC65 were reformulated, and they were not quantitatively validated in a new cohort. The relevance and consistency of the reformulated items were validated only by an expert group. The objective of this study was to validate the IPC65 in a new cohort of healthcare professionals working in interdisciplinary teams.

## Methods

### Theoretical framework

The various definitions of interdisciplinarity have in common the indication that an interdisciplinary team is composed of members from various disciplines. These members are interdependent, have collaborative roles and meet to share information in order to effectively provide quality patient-centered care [5,8]. Interdisciplinary teams thus aim to improve integration in the care process. The definition of integration by Contandriopoulos et al. [14] is as follows: “the process of organizing sustainable consistency, over time, between a system of values, an organizational structure and a clinical system so as to create a space in which stakeholders (individuals and organizations concerned) find it meaningful and beneficial to coordinate their practices within a particular context”. According to these authors, the integration process aims to establish cooperative relationships in situations of interdependence with the common objective of incorporating the following 5 dimensions: normative integration, functional integration, clinical integration, care integration and systemic integration.

**Fig 1:**
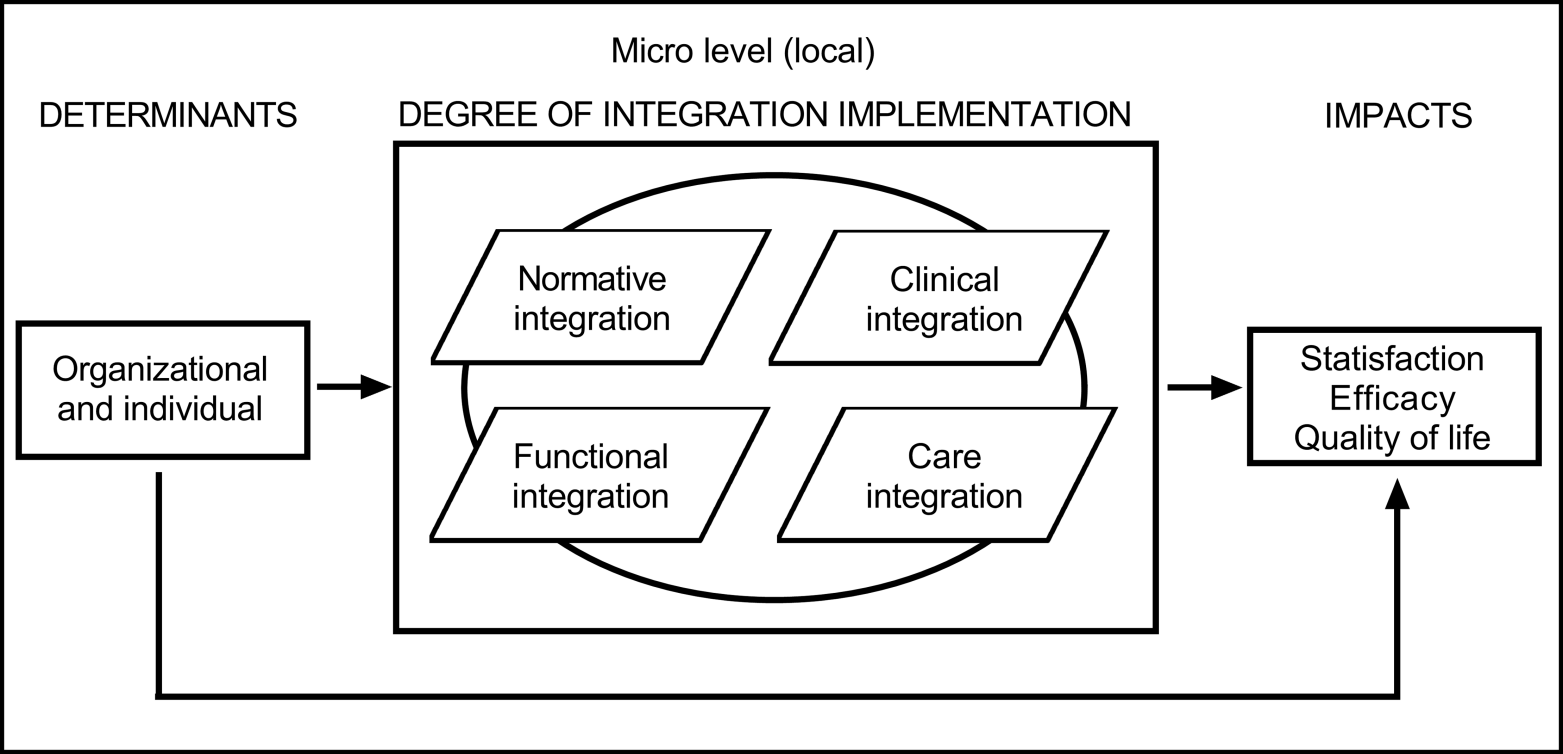
Conceptual framework for the analysis of integration implementation in the healthcare process, taken from Roberge et al. [15] and adapted from the model by Contandriopoulos et al. [14].

In the context of interdisciplinarity, **normative integration** consists of having a clear vision of team goals, a common interest in interdisciplinary work and leadership in the team that allows for good teamwork to achieve its objectives. **Functional integration** consists of having administrative support to gain the resources necessary for good clinical functioning. **Clinical integration** aims to clearly define the role of each professional, manage meetings, and establish work rules and mechanisms to resolve conflicts. **Care integration** aims to coordinate clinical practices related to the team structure and operations in order to improve the quality of care and patient services. **Systemic integration** concerns the interaction between the local system of interdependent professionals (i.e., the hospital) and the environment in which it is located (i.e., the healthcare system). Based on Roberge et al. [15], only the first 4 dimensions were considered in the creation of the evaluation tool for measuring the integration of local interdisciplinarity with the aim of improving daily practice (Fig 1).

### Description of the questionnaire

The details of the process of creating and validating the initial version of the questionnaire are described elsewhere [13]. The IPC65 questionnaire is actually in French Canadian. The questionnaire measures 4 of the 5 dimensions of care process integration theorized by Contandriopoulos et al. [14], and it consists of 65 items grouped into 12 sub-dimensions representing specific aspects of integration. The details related to the sub-dimensions and the number of items by dimension and sub-dimension are presented in Table 1. Integration is measured with a Likert scale of 4 levels ranging from totally agree (score = 3) to totally disagree (score = 0) with an option to choose not applicable. An even number of levels was chosen to compel the respondent to take a positive or negative position [16]. Most items are worded using a positive structure, but some are not to avoid acquiescence bias [17]. A total score and a score for each dimension can be calculated using the means of items (i.e. sum of all scores divided by the number of items). To note that items with negative structures must be reversed to do so. Items in which “not applicable” is selected are not considered. A score above 2.5 indicates very good integration of the concepts leading to interdisciplinarity; a score between 2 and 2.5 indicates good integration; a score between 1 and 2 indicates differences or average integration, and a score of 1 or less indicates poor integration.

**Table 1:** Dimensions and sub-dimensions of IPC65 and IPC59.

Dimensions and sub-dimensions	# items IPC65	# items IPC59	Change from IPC65 to IPC59		
			Displaced		Eliminated
Normative integration	10	10	0	0	
Vision	2	2			
Interest in interdisciplinarity	5	5			
Leadership	3	3			
Functional integration	9	8	2	1	
Administrative support	2	4	2		
Resources available	7	4		1	
Clinical integration	26	23	2	3	
Explicit formalization of roles	4	3	1		
Meeting management^1^	5	-		1	
Working rules^1^	8	-	1	1	
Internal functioning and conflict resolution mode	9	10		1	
Meeting management and working rules	-	10			
Care integration	20	18	0	2	
Results related to the structure	6	5		1	
Results related to the team	6	6			
Results related to patients	8	7		1	

^1^ These two sub-dimensions were regrouped in IPC59: “Meeting management and working rules”

### Data collection

To complete the survey, participants had to be over 18 years old and be working in an interdisciplinary team in a healthcare institution in Quebec, Canada. Questionnaires were distributed in paper or electronic format by managers in healthcare institutions and presidents of local multidisciplinary councils. In addition to the 65 questions on interdisciplinarity, the name of the institution, the healthcare domain, the number of professionals in the team, the profession of the respondent and the number of years of experience in interdisciplinarity were collected. To help the respondent understand the actual meaning of interdisciplinarity, definitions of interdisciplinary teams, multidisciplinary teams and interdisciplinary intervention plans were provided on the presentation page. The questionnaire was completed anonymously, and subjects’ responses corresponded to their provision of consent. The Ethics Review Board of the CHUS (Comité d'éthique de la recherche en santé chez l'humain du CHUS) approved the study.

### Statistical analysis

This study was conducted to validate the new version of the questionnaire with 65 items instead of 99 items as in the initial version. The validation was conducted to evaluate the psychometric qualities of the questionnaire and determine whether this version could have been further improved. Similar analyses to those conducted for external quantitative validation in the study by Bédard et al. [13] were performed.

The first step was to evaluate the response rate for each item with descriptive statistics. Items with response rates less than 85% or with rates of “not applicable” responses above 10% were identified. Then, internal consistency was measured with Cronbach’s alpha values for the entire questionnaire and each dimension. A value greater than 0.7 was recommended [18]. The same analyses were performed by excluding one item at a time. If Cronbach’s alpha values increased by more than 2%, the item was considered potentially problematic. Inter-item correlation was measured with Pearson’s correlation coefficients among all items within a dimension. Correlation coefficients higher than 0.75 suggested redundancy. Pearson’s correlation coefficients were also used to evaluate the consistency between each item and the dimension score. In this case, items with correlations less than 0.4 were considered problematic [18].

Construct validity was evaluated with a principal component analysis (PCA) with varimax rotation for each dimension [19–20]. The aim of this analysis was to determine and potentially reduce/regroup the number of independent sub-dimensions in the questionnaire. Graphical representations of PCA can help understand data structure by focusing on the variability explained by different groups of variables. The first component or eigenvalue corresponds to a composite score that maximizes the proportion of variance explained by the variables included in the dimension. All components are independent and explain the maximum amount of variance among the unexplained variability in the previous component. In this study, eigenvalues ≥ 0.8 were used to create a matrix with varimax rotation and obtain a minimum of 2 components per dimension. The weight attributed to each item helped identify items with a low contribution to the variance. Items with a weight above 0.40 were considered good for a component explaining more than 20% of the total variability. For a component explaining less than 20% of the variability, items were considered good if their contribution was above 0.70. Graphical representations of the first two components were built to evaluate whether items could be grouped within the same sub-dimension or needed to be moved into another sub-dimension.

The sample size required to conduct the analysis was calculated according to the rule of Hatcher [21] who recommended that the number of subjects should be larger of 5 times the number of variables. In this study, this corresponded to 325. All analyses were performed with SPSS software version 23 (IBM, New York, USA), and figures were created with GraphPad version 6.

## Results

### Participants

Between January 2013 and June 2014, 398 participants across 12 cities in Quebec responded to the survey. Five questionnaires were eliminated because less than 50% of the questions had been answered, and 1 respondent answered “not applicable” on all questions. Of the 392 questionnaires included in the analysis, 342 (87.2%) were 100% complete without missing data. Of the total number of questions (i.e., 392 questionnaires with 65 questions), only 0.3% were missing data, and 2.9% received responses of “not applicable”. Half of the surveys were completed online (i.e., 48.5%). The median amount of time to complete each questionnaire was 12 minutes. The completion time could only be calculated for online questionnaires since the software noted the respondent’s start and end time. Professionals from 57 different teams with a median of 4 respondents per team responded to the survey. The respondents came from 16 healthcare institutions, with the majority of participants working in health and social service centers (32.7%) and rehabilitation centers (31.6%).

### Validation

**Fig 2:**
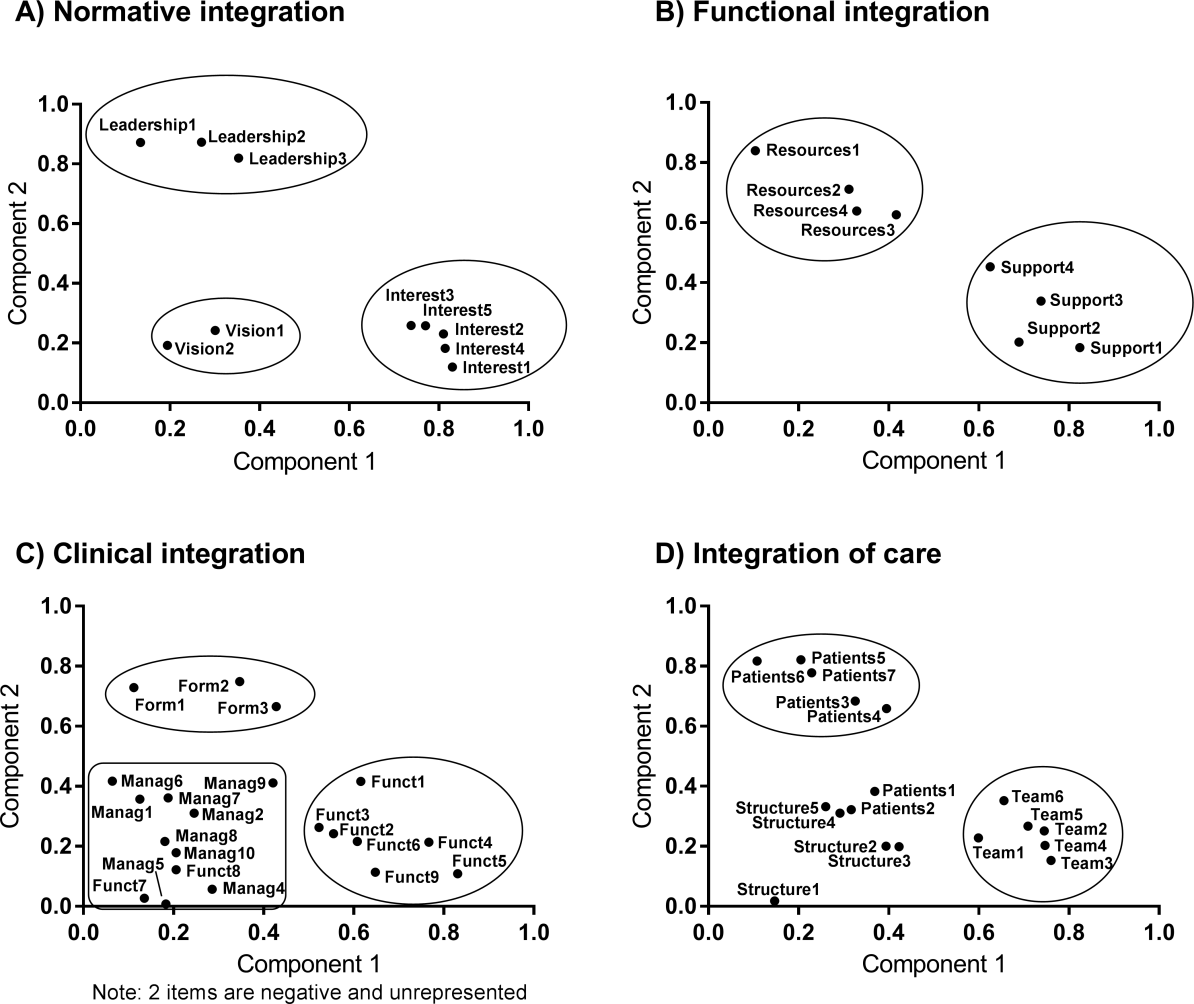
Graphical representation of principal component analysis for each dimension of integration.

In this version of the tool with 65 items, no change was necessary for the dimension “normative integration”. This dimension had the best psychometric properties with 96.7% complete surveys (i.e., without missing data or “not applicable” responses) and a very good Cronbach’s alpha (α = 0.904). Only two items had high inter-item correlation, but as they measured different concepts, it was decided to retain both of them. According to the PCA, 3 components had eigenvalues ≥ 0.8 and explained 77.7% of the variability. Items from the 3 sub-dimensions were clearly grouped together when considering the PCA graphical representation (Fig 2, part A).

The dimension of “functional integration” had good properties, including 91.4% complete surveys, a good Cronbach’s alpha (α = 0.872) and only one problematic item according to the PCA. One item related to the integration of trainees received many “not applicable” responses (8.9%) and was highly correlated with the integration of new professionals (r = 0.686). This item was eliminated. Another PCA analysis without this item explained 61.1% of the variability with 2 eigenvalues ≥ 0.8. The PCA graph suggested that 2 items in the resources sub-dimension were more associated with the support sub-dimension. After discussion, it was decided to displace these two items to the support sub-dimension. The phrasing of these two items was slightly modified for more coherence.

**Fig 3:**
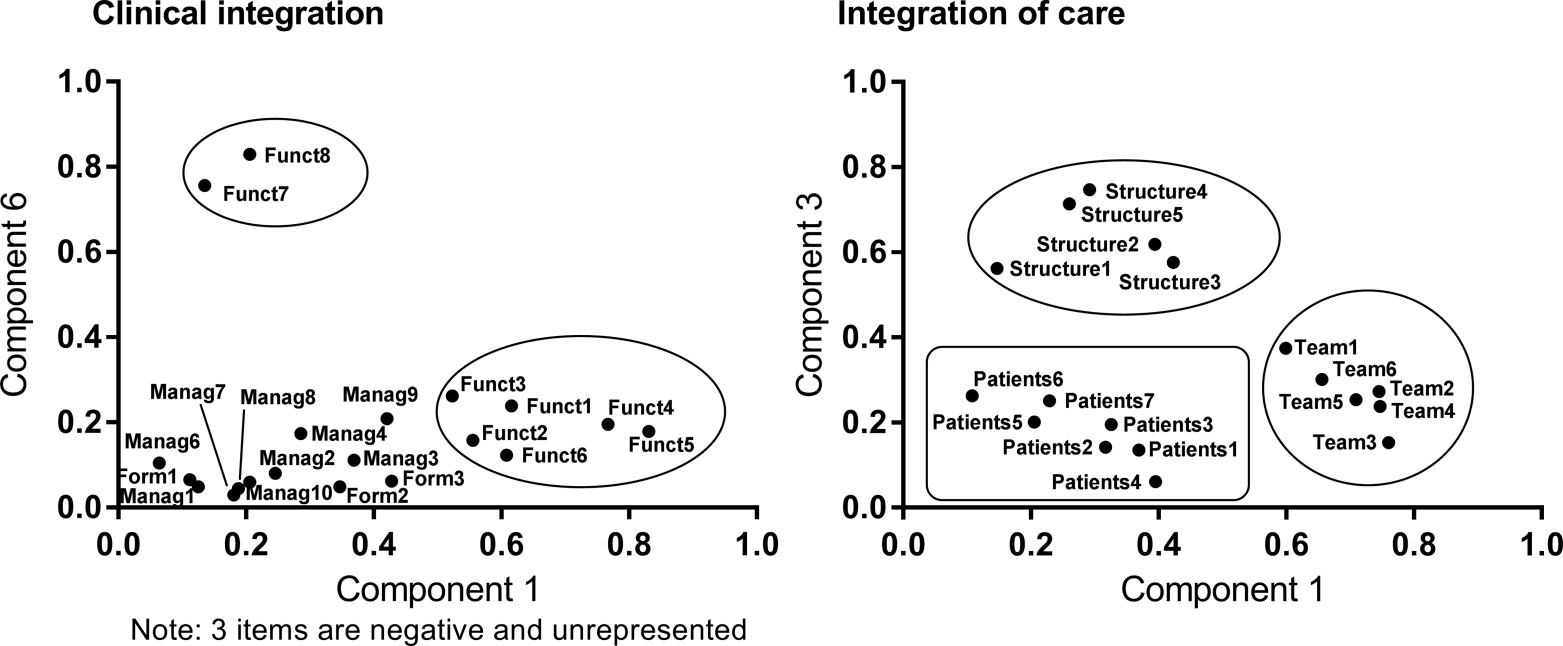
Graphical representation of principal component analysis for new sub-dimensions of clinical integration and integration of care.

The dimension “clinical integration” was more problematic and included 26 items. The Cronbach’s alpha was very good (α = 0.923), but there were different problems with the items in this dimension. Two items in the internal functioning sub-dimension received a nearly 20% rate of “not applicable” responses. These items weakly correlated with the dimension score (r = 0.176 and r = 0.426), and one item did not show good contribution in the PCA. One of these items was thus eliminated, as well as two others since they seemed to be redundant with other items. The exclusion of these 3 items increased the number of complete surveys (from 56.4% to 60.2%) and reduced the number of items with low contribution in the PCA (8 to 4). Another PCA analysis performed for this dimension with the remaining items yielded 7 eigenvalues ≥ 0.8 explaining 70.3% of the variability. In Fig 2 (part C), the majority of “internal functioning” items were grouped together, and one item from the “working rules” sub-dimension and one from the “formalization” sub-dimension were grouped and displaced to this sub-dimension. According to the PCA, items from the “working rules” and “meeting management” sub-dimensions seemed to explain the same component and were merged to form a new sub-dimension “meeting management and working rules”. Finally, two items in the “internal functioning” sub-dimension were different from the other items in this sub-dimension and were grouped only on the sixth component of the PCA (Fig 3). Since these items were considered important and were logically related to this sub-dimension, it was decided to keep them in their initial sub-dimension (Funct7 and Funct8 in Fig 2, part C).

In the “care integration” dimension and “structure” sub-dimension, one item received a 13% rate of “not applicable” responses and was eliminated. In addition, this item could be considered as a sub-item of another item. In the sub-dimension relatives of patients, two items were highly correlated (r = 0.787) and were reformulated to create a unique item. From the PCA analysis, 4 eigenvalues were ≥ 0.8 and explained 69.7% of the variability. In the PCA graph (Fig 2, part D), all items of the team sub-dimension were clearly grouped together, and the majority of items in the patients sub-dimension were associated except for 2 items. By analyzing the third component of the PCA, it was clear that it was explained by items of the structure sub-dimension, and a new graph with components 1 and 3 was created (Fig 3). In this new graph, all items of all 3 sub-dimensions were grouped separately, and it was decided to keep items in their respective sub-dimensions.

After this new validation of our questionnaire, the IPC59 was defined with six items that were eliminated from the IPC65, and two sub-dimensions were merged. Four items were moved to another sub-dimension, and 8 items were very slightly reworded. However, 17 items continued to be potentially problematic (1 item with an 18.4% rate of not applicable responses, 6 with correlations > 0.75, and 10 with PCA ≤ 0.4/0.7) (Table 2). For the 6 items with high inter-item correlation, there was no redundancy since all items measured different concepts. For the 10 items with PCA values ≤ 0.4/0.7, 7 had PCA values between 0.6–0.7, and the 3 remaining had values between 0.5–0.6, which were still high. Also note that the number of complete surveys is higher with IPC59 than IPC65 with an increase from 155 to 179 complete surveys. This was mainly due to the elimination of items having a low rate of answers (i.e., items with at least 15% of “missing data” or 10% of “not applicable”). This implies that the new version with 59 items is more in line with the reality of professionals involved in interdisciplinary teams since a large majority of items are meaningful for them. Considering the higher number of complete surveys in each dimension in comparison to this number in the full questionnaire, this simply indicates a high dispersion of “missing data” and “not applicable” across the items, thus reflecting a high heterogeneity in what is potentially meaningless for respondents.

**Table 2:** Descriptive statistics before and after validation of the questionnaire (n = 392).

	Total	Normative integration	Functional integration	Clinical integration	Care integration
**Before statistical validation**					
Number of items	65	10	9	26	20
Complete survey ^a^	155 (39.5%)	379 (96.7%)	319 (81.4%)	221 (56.4%)	271 (69.1%)
Items without answer ^b^	3	0	0	2	1
Cronbach’s alpha (CA)	0.969	0.904	0.872	0.923	0.947
Inter-item correlations > 0.75	8	2	0	4	2
Items with 2% increase of CA	0	0	0	0	0
Correlation items-dimension ≤ 0.4	2	0	0	1	0
Items with PCA ≤ 0.4/0.7 ^c^	-	0	1	8	8
**After statistical validation**					
Number of items	59	10	8	23	18
Complete survey ^a^	179 (45.7%)	379 (96.7%)	349 (89.0%)	236 (60.2%)	296 (75.5%)
Items without answer ^b^	1	0	0	1	0
Cronbach’s alpha (CA)	0.967	0.904	0.857	0.918	0.943
Inter-item correlations > 0.75	6	2	0	4	0
Items with 2% increase of CA	0	0	0	0	0
Correlation items-dimension ≤ 0.4	1	0	0	1	0
Items with PCA ≤ 0.4/0.7 ^c^	-	0	1	4	5

^a^: Questionnaires without “missing data” or without “not applicable”

^b^: Items with at least 15% of “missing data” or 10% of “not applicable”

^c^: When the variability of the component was higher or equal to 20%, number of items with a score ≤ 0.4 (≤0.7 if variability was less than 20%).

## Discussion

The statistical validation conducted in this study demonstrates the good psychometric properties of the questionnaire. In particular, the validation of the questionnaire with 65 items provided better results than the validation of the initial version with 99 items. This was mainly due to the number of completed questionnaires and the number of items with correlations higher than 0.75 that dropped from 46 to 8. However, the Cronbach’s alpha values were very good for all versions of the questionnaire and above 0.85 for all dimensions. The version composed of 99 items was improved for the IPC65 and IPC59, where the number of potentially problematic items decreased from 52% to 34% and 28.8%, respectively. The discrimination of items that were considered redundant, ambiguous or less important improved the content and validity of the questionnaire. The IPC65 and IPC59 had similar properties, but the PCA graphical representations were clearly better for the IPC59 than the IPC65 where all items of each sub-dimension were regrouped together. All items considered potentially problematic according to the statistical criteria in our study were analyzed to evaluate if their exclusion could have improved the questionnaire. After a thorough analysis and discussion, the statistical issues found for these items were not considered critical, and they were retained since each item measured different concepts and elements that were important for measuring good functioning of interdisciplinarity.

One of the strengths of this study is the rigorous approach used to establish and validate the components of interdisciplinarity in healthcare practice. All steps of the validation process improved the questionnaire by reducing the number of items, reformulating ambiguous items and more appropriately classifying them in the appropriate sub-dimensions. Also, the IPC59 covers a wide range of dimensions in interdisciplinary, which is essential considering the numerous implications of this approach in healthcare and that many conditions are necessary to make it work [2,10,22]. Different from a previous work developed in a long-term care setting [23], the IPC59 was developed in a context of short-term care and shows higher internal consistency.

Our study also has some limitations. The first one is the length of the questionnaire, which could limit its use. To facilitate the completion of the questionnaire, the number of items was reduced from 99 to 65 and then to 59 during the quantitative validation. Additionally, the response rate was good (i.e., 361/392 (92%), with participants responding to 90% or more of the questions on the IPC65), and the median time to respond was 12 minutes for 65 questions, which was reasonable. Another limit is related to the fact that we used a convenience sample. This may have influenced our results.

## Conclusion

The IPC59 showed good reliability and validity. This tool will help healthcare managers and professionals identify the strengths and weaknesses of their interdisciplinary team and ensure its continuous improvement. By identifying the degree of integration of key concepts in interdisciplinarity, healthcare managers and professionals will be able to develop new strategies to reinforce their collaboration for the benefit of patients.

## Supporting information

S1 File Version 59 items 2016. (DOC)
