# Supplementary material for: Measuring interdisciplinarity in clinical practice with IPC59, a modified and improved version of IPC65
Source: PLoS One. 2018 Jul 6;13(7):e0197484. doi: 10.1371/journal.pone.0197484 (PMC6034788; doi:10.1371/journal.pone.0197484)
Supplement: S1 File — (DOC) [file pone.0197484.s001.doc]

| **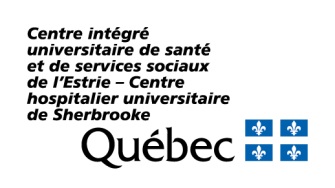** | **Évaluation de l’interdisciplinarité en pratique clinique (Questionnaire IPC59)** |
| --- | --- |

Ce questionnaire anonyme est à compléter par tout **intervenant** travaillant dans une **équipe interdisciplinaire en santé.** Son objet est de **mesurer le degré d’intégration d’une équipe interdisciplinaire** par rapport aux standards établis dans la littérature scientifique.

**Définition :**  Une **équipe interdisciplinaire** consiste en un « regroupement de plusieurs intervenants ayant une formation, une compétence et une expérience spécifique qui travaillent ensemble à la compréhension globale, commune et unifiée d’une personne en vue d’une intervention concentrée à l’intérieur d’un partage complémentaire des tâches. L’interdisciplinarité exige une synthèse et une harmonisation entre les points de vue qui s’intègrent en un tout cohérent et coordonné. » Hébert (1997).

**Définition** **:** Une **équipe multidisciplinaire** consiste en un « regroupement de plusieurs intervenants où chaque professionnel accomplit sa tâche de façon indépendante. Chacun représente sa propre discipline, fait ses propres évaluations, fixe ses propres objectifs, rédige ses rapports, implante sa programmation et en assure le suivi. Il décide seul de ce qui est bon pour le client. Il établit un plan d’intervention et en assume la responsabilité. » (Larivière et Ricard, 1998).

**Définition** **:** Le **plan d’intervention interdisciplinaire** consiste en un **«**ensemble d’interventions planifiées conjointement par les membres de l’équipe interdisciplinaire en vue de satisfaire les besoins de soins et d’assistance du client au cours d’un épisode de soins intra-établissement et/ou inter-établissement. » (Leprohon et Lessard, 2006, p.18).

**Nom de votre établissement**

**Domaine de votre équipe interdisciplinaire**

**Nombre d’intervenants dans votre équipe**

**Nommez votre profession**

**Nombre d’années d’expérience en interdisciplinarité**

**Important : Répondez spontanément à partir de la situation réelle dans votre équipe interdisciplinaire et non pas en considérant la situation que vous souhaiteriez avoir.**

| **Intégration normative** | **Cotation** | | | | | | | | |
| --- | --- | --- | --- | --- | --- | --- | --- | --- | --- |
| Éléments à considérer   - Vision - Intérêt pour le travail en interdisciplinarité - Leadership | Totalement en accord | Plutôt en accord | | Plutôt en désaccord | | Totalement en désaccord | | Non applicable | |
| *Vision* | | | | | | | | | |
| Il existe une vision claire des objectifs au sein de l’équipe : | | | | | | | | | |
| 1. *Travailler en synergie afin de prodiguer des soins et services de qualité qui répondent aux besoins de la population cible* |  | |  | |  | |  | |  |
| 1. *Contribuer à assurer un meilleur continuum de soins et service pour la population cible* |  | |  | |  | |  | |  |
| *Intérêt pour le travail en interdisciplinarité* | | | | | | | | | |
| 1. Tous les membres de l’équipe croient aux bénéfices du travail en Interdisciplinarité |  | |  | |  | |  | |  |
| 1. Tous les membres de l’équipe se sentent impliqués dans le bon fonctionnement de l’équipe |  | |  | |  | |  | |  |
| 1. Le rôle de chacun est considéré utile au bon fonctionnement de l’équipe et à l’atteinte de soins de qualité |  | |  | |  | |  | |  |
| 1. La motivation à travailler en groupe est perçue par les membres comme étant importante |  | |  | |  | |  | |  |
| 1. Les interactions se font dans un climat de confiance et de respect mutuel |  | |  | |  | |  | |  |
| *Leadership* | | | | | | | | | |
| 1. Il existe un leadership médical et/ou clinico-administratif au sein de l’équipe |  | |  | |  | |  | |  |
| 1. Le leadership exercé permet à l’équipe de bien fonctionner et d’atteindre ses objectifs |  | |  | |  | |  | |  |
| 1. Le leadership exercé permet de créer un climat de confiance en favorisant la communication et la transparence |  | |  | |  | |  | |  |

| **Intégration fonctionnelle** | **Cotation** | | | | | | | | |
| --- | --- | --- | --- | --- | --- | --- | --- | --- | --- |
| Éléments à considérer   - Appui administratif - Ressources disponibles | Totalement en accord | Plutôt en accord | | Plutôt en désaccord | | Totalement en désaccord | | Non applicable | |
| *Appui administratif* | | | | | | | | | |
| 1. L’établissement reconnaît l’importance de l’équipe interdisciplinaire |  | |  | |  | |  | |  |
| 1. L’établissement favorise et soutient une composition adéquate de l’équipe dans le but de fournir des soins et services de qualité |  | |  | |  | |  | |  |
| 1. L’établissement soutient les membres de l’équipe dans leur processus d’amélioration continue de la qualité des soins et des services offerts à la population cible |  | |  | |  | |  | |  |
| 1. L’établissement fournit un soutien administratif au bon fonctionnement de l’équipe |  | |  | |  | |  | |  |
| *Ressources disponibles* | | | | | | | | | |
| Le service dont dépend l’équipe fournit les ressources nécessaires : | | | | | | | | | |
| 1. *Formation visant à rehausser les compétences de chacun* |  | |  | |  | |  | |  |
| 1. *Coaching (au besoin) en interdisciplinarité* |  | |  | |  | |  | |  |
| 1. *Intégration des personnes nouvellement enrôlées* |  | |  | |  | |  | |  |
| 1. La stabilité de l’équipe et la rétention du personnel sont encouragées (reconnaissance, promotions, etc.) |  | |  | |  | |  | |  |

| **Intégration clinique** | **Cotation** | | | | | | | | |
| --- | --- | --- | --- | --- | --- | --- | --- | --- | --- |
| Éléments à considérer   - Formalisation explicite des rôles et partage des tâches - Gestion des réunions et règles de travail - Fonctionnement interne et mode de résolution des conflits | Totalement en accord | Plutôt en accord | | Plutôt en désaccord | | Totalement en désaccord | | Non applicable | |
| *Formalisation explicite des rôles et partage des tâches* | | | | | | | | | |
| 1. Les rôles et tâches de chacun sont bien définis et formellement documentés en conformité avec les lois 90 et 21 |  | |  | |  | |  | |  |
| Le partage des tâches est optimal en ce qui concerne : | | | | | | | | | |
| 1. *Les soins et les services prodigués au patient* |  | |  | |  | |  | |  |
| 1. *Le fonctionnement interne de l’équipe* |  | |  | |  | |  | |  |
| *Gestion des réunions et règles de travail* | | | | | | | | | |
| Les réunions permettent concrètement de : | | | | | | | | | |
| 1. *Générer des objectifs communs vis-à-vis de l’état de santé du patient* |  | |  | |  | |  | |  |
| 1. *Coordonner les actions des différents intervenants* |  | |  | |  | |  | |  |
| 1. Lorsque requis, les membres de l’équipe sont présents aux réunions interdisciplinaires |  | |  | |  | |  | |  |
| 1. La fréquence des réunions de l’équipe interdisciplinaire est suffisante pour permettre une intercommunication et une collaboration active entre les membres de l’équipe |  | |  | |  | |  | |  |
| 1. Il existe également des réunions sur les processus de fonctionnement |  | |  | |  | |  | |  |
| 1. Le plan d’intervention intègre de façon harmonieuse les interventions des différentes disciplines et/ou spécialités |  | |  | |  | |  | |  |
| 1. Les documents utiles au bon fonctionnement de l’équipe (documents d’encadrement clinique, protocoles de soins, plan d’intervention, etc.) sont connus, à jour et disponibles |  | |  | |  | |  | |  |
| 1. Les canaux formels et informels sont utilisés pour informer les membres de l’équipe |  | |  | |  | |  | |  |
| 1. Les règles de fonctionnement de l’équipe sont bien comprises |  | |  | |  | |  | |  |
| 1. Des mécanismes d’amélioration continue du fonctionnement de l’équipe sont utilisés (révision des processus d’intervention, remise en question et discussion franche sur le fonctionnement, etc.) |  | |  | |  | |  | |  |
| *Fonctionnement interne et mode de résolution des conflits* | | | | | | | | | |
| 1. Le rôle de chacun est interdépendant et collaboratif, fondé sur une collaboration interprofessionnelle mature |  | |  | |  | |  | |  |
| 1. L’autonomie professionnelle de chacun est reconnue |  | |  | |  | |  | |  |
| 1. La communication entre les membres est claire : chacun comprend ce que l’autre veut dire et utilise la rétroaction pour clarifier les situations |  | |  | |  | |  | |  |
| 1. Les membres de l’équipe ont une bonne capacité de remise en question (introspection, ouverture d’esprit, etc.) |  | |  | |  | |  | |  |
| 1. Les membres de l’équipe font preuve de maturité dans la résolution de leurs différends |  | |  | |  | |  | |  |
| 1. Les conflits associés au mode de fonctionnement en interdisciplinarité sont normaux et peuvent être résolus |  | |  | |  | |  | |  |
| 1. Les conflits associés au mode de fonctionnement en interdisciplinarité sont fréquents |  | |  | |  | |  | |  |
| 1. Les logiques individuelles et professionnelles de certains membres de l’équipe peuvent miner la collaboration en interdisciplinarité |  | |  | |  | |  | |  |
| 1. Les membres de l’équipe sont solidaires face aux critiques externes émises à l’encontre des décisions prises en interdisciplinarité |  | |  | |  | |  | |  |
| 1. Les médecins perçoivent davantage les autres professionnels comme étant à leur service et non comme des collaborateurs à part entière |  | |  | |  | |  | |  |

| **Intégration des soins** | **Cotation** | | | | | | | | |
| --- | --- | --- | --- | --- | --- | --- | --- | --- | --- |
| Éléments à considérer   - Ce qui vise la structure - Ce qui vise l’équipe - Ce qui vise le patient | Totalement en accord | Plutôt en accord | | Plutôt en désaccord | | Totalement en désaccord | | Non applicable | |
| *Résultats relatifs à la structure* | | | | | | | | | |
| Le mode de fonctionnement de votre équipe permet : | | | | | | | | | |
| 1. *De bien planifier l’allocation des ressources (la bonne personne au bon moment)* |  | |  | |  | |  | |  |
| 1. *D’assurer la cohérence, la qualité et la continuité des soins et des services dispensés par l’établissement* |  | |  | |  | |  | |  |
| 1. *De donner aux équipiers le sentiment d’être utile, de pouvoir influer sur la capacité opérationnelle de l’organisation* |  | |  | |  | |  | |  |
| 1. *De favoriser le recours à des outils d’évaluation des besoins des patients et de leurs familles* |  | |  | |  | |  | |  |
| 1. *De favoriser la collaboration avec les partenaires externes (médecin de famille, SAAQ, CSST, établissements de santé, etc.)* |  | |  | |  | |  | |  |
| *Résultats relatifs à l’équipe* | | | | | | | | | |
| Le mode de fonctionnement de votre équipe : | | | | | | | | | |
| 1. *Permet de traiter des cas complexes (dont le diagnostic et le traitement demandent l’expertise et le jugement de plusieurs cliniciens expérimentés)* |  | |  | |  | |  | |  |
| 1. *Permet de développer davantage les connaissances et les habiletés d’intervention (compétences professionnelles)* |  | |  | |  | |  | |  |
| 1. *Permet le soutien mutuel et facilite l’intégration des débutants et des stagiaires par des professionnels plus expérimentés (mentorat, préceptorat)* |  | |  | |  | |  | |  |
| 1. *Favorise le respect et la reconnaissance de la spécificité de chacun* |  | |  | |  | |  | |  |
| 1. *Permet de transcender ses valeurs personnelles pour travailler au partage de valeurs professionnelles communes* |  | |  | |  | |  | |  |
| 1. *Permet de rassurer l’équipe sur la pertinence des interventions* |  | |  | |  | |  | |  |
| *Résultats relatifs aux patients* | | | | | | | | | |
| Le mode de fonctionnement de votre équipe permet : | | | | | | | | | |
| 1. *L’atteinte des objectifs cliniques (guérison, maintien ou amélioration des capacités fonctionnelles et cognitives, retour au travail, etc.)* |  | |  | |  | |  | |  |
| 1. *Des services sécuritaires (respect des normes et des pratiques organisationnelles requises pour l’agrément)* |  | |  | |  | |  | |  |
| 1. *De mieux cibler les besoins et les attentes du patient et/ou de sa famille* |  | |  | |  | |  | |  |
| Le mode de fonctionnement de votre équipe favorise, au cours de l’épisode de soins : | | | | | | | | | |
| 1. *La transmission d’un message cohérent et uniforme au patient et/ou à sa famille* |  | |  | |  | |  | |  |
| 1. *L’adhésion du patient au plan d’intervention et l’implication de la famille* |  | |  | |  | |  | |  |
| 1. *L’implication du patient dans son processus de guérison (affirmation de soi / empowerment)* |  | |  | |  | |  | |  |
| 1. *L’accompagnement du patient et de sa famille* |  | |  | |  | |  | |  |

**Merci de votre aimable participation**

Comment citer ce questionnaire :

Poder T.G., Carrier N. et Bédard S.K. (2016).

Contacts :

Thomas G. Poder

Conseiller-cadre en ETMIS

Chercheur au CRCHUS

CIUSSS de l’Estrie - CHUS

[tpoder.chus@ssss.gouv.qc.ca](mailto:tpoder.chus@ssss.gouv.qc.ca)

**Comment utiliser ce questionnaire ?**

Ce questionnaire sert principalement à évaluer une équipe dans son ensemble. Différents scores du degré d’intégration des concepts menant à une pratique interdisciplinaire peuvent être calculés. Un score global peut ainsi être calculé pour indiquer un résultat d’ensemble, de même qu'un score par sous-dimension et un score pour chaque question pour indiquer des résultats spécifiques. Au niveau des sous-dimensions et des questions individuelles, les scores obtenus permettent d’évaluer les points forts et les points faibles d’une équipe. Éventuellement, il sera aussi possible d’observer des points de divergences au sein de l’équipe pour certaines questions.

Chaque réponse peut être transformée en un score compris entre 0 et 3 ou 0 correspond à « totalement en désaccord », 1 à « plutôt en désaccord », 2 à « plutôt en accord » et 3 à « totalement en accord ». Les réponses « non applicable » sont exclues du calcul du score. Dans le calcul du score, aucune pondération n’est effectuée, chaque item étant considéré comme également important.

Un score entre 2,5 et 3 indique une bonne intégration des concepts menant à une pratique interdisciplinaire efficace. Pour un item individuel cela correspond à un point fort.

Un score entre 2 et 2,5 indique un fonctionnement assez bon avec quelques éléments d'amélioration à apporter après examen en groupe.

Un score entre 1 et 2 indique soit des divergences marquées, soit plusieurs situations qu'il faudrait examiner en groupe et potentiellement corriger.

Un score inférieur à 1 indique qu'il y a très vraisemblablement un ou plusieurs problèmes à régler. Pour un item individuel cela correspond à un point faible.
